# The Evolution of Adenoviral Vectors through Genetic and Chemical Surface Modifications

**DOI:** 10.3390/v6020832

**Published:** 2014-02-17

**Authors:** Cristian Capasso, Mariangela Garofalo, Mari Hirvinen, Vincenzo Cerullo

**Affiliations:** 1 Laboratory of Immunovirotherapy, Division of Pharmaceutical Biosciences and CDR, Faculty of Pharmacy, University of Helsinki, Biocenter 2, Viikinkaari 5, Helsinki 00790, Finland; E-Mails: cristian.capasso@helsinki.fi (C.C.); mari.hirvinen@helsinki.fi (M.H.); 2 Department of Molecular Medicine and Medical Biotechnology, University of Naples “Federico II”, Via S.Pansini 5, Naples 80131, Italy; E-Mail: mariangela.garofalo@helsinki.fi

**Keywords:** gene therapy, adenoviral vectors, genetic modification, chimeric fibers, surface modification, pseudotyping, polymers, immunogenicity, re-targeting

## Abstract

A long time has passed since the first clinical trial with adenoviral (Ad) vectors. Despite being very promising, Ad vectors soon revealed their limitations in human clinical trials. The pre-existing immunity, the marked liver tropism and the high toxicity of first generation Ad (FG-Ad) vectors have been the main challenges for the development of new approaches. Significant effort toward the development of genetically and chemically modified adenoviral vectors has enabled researchers to create more sophisticated vectors for gene therapy, with an improved safety profile and a higher transduction ability of different tissues. In this review, we will describe the latest findings in the high-speed, evolving field of genetic and chemical modifications of adenoviral vectors, a field in which different disciplines, such as biomaterial research, virology and immunology, co-operate synergistically to create better gene therapy tools for modern challenges.

## 1. Introduction

Gene therapy represents a modern and fascinating approach to science and it has been considered the solution to several monogenic diseases. The simple idea of gene replacement, by delivering a functional copy of the mutated gene, seems to be the answer to a wide variety of genetic disorders, such as muscular dystrophy, phenylketonuria or adenosine deaminase severe combined immunodeficiency (ADA-SCID). 

The first clinical trial in human, with cells engineered with a virus, was performed in 1990 by Rosenberg and colleagues. They re-infused autologous tumor infiltrating lymphocytes (TILs) *ex vivo* infected with a retrovirus in patients with melanoma [1]. However, a decade later, the first death of a human patient, due to an excessive immune reaction to the Ad vector, alerted the scientific community and slowed down the progress in the field [2]. This unfortunate event raised doubts about the knowledge of vectors for gene therapy and, as a result, much more resources have been invested to comprehend the biology and the immunological aspects of vector-host interactions. 

Ad vectors show efficient transduction *in vitro*, a good ability to infect dividing and non-dividing cells (*in vitro* and *in vivo*) and the possibility of inserting big expression cassettes, as well as producing high titers of vector of good manufacturing practice (GMP) quality. For these reasons, Ad vectors represent the most used viral vectors in gene therapy clinical trials (23.3%) [3]. Due to their outstanding potential, adenoviral vectors have been engineered and modified in order to improve the transduction efficiency and, above all, the safety profile. 

### Immunology of Ad Vectors

**Figure 1:**
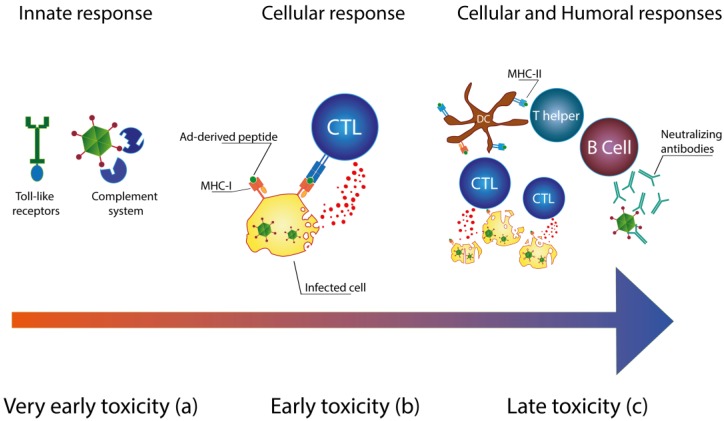
Immune response to Ad vectors. (**a**) Activation of the innate immune system leads to the very early toxicity. Toll-like receptors (TRLs) and the complement system are responsible for the induction of pro-inflammatory cytokines; (**b**) A few days after the administration of the Ad vector, CTLs recognize Ad-derived peptides on MHC-I molecules. Activation of CTLs results in the lysis of infected cells; (**c**) Mature antigen presenting cells (APCs) can cross-present Ad-derived peptides on the major histocompatibility complex (MHC)-I, enhancing the cytotoxic activity of CTLs. In addition, T-helper lymphocytes are primed by APCs that present Ad-derived peptides on MHC-II. Mature T-helper lymphocytes stimulate B-lymphocytes leading to the production of neutralizing antibodies (NAbs).

The death of a patient in 1999 during a gene therapy clinical trial revealed one of the main characteristics of Ad vectors: the immunogenicity of adenoviruses [4]. *In vivo* toxicity of Ad vectors is triggered by three different mechanisms: innate immune response, cellular response and humoral response (Figure 1) [5]. The activation of the innate immune system results in the very early toxicity. Pattern recognition receptors (PRRs) [6,7] and the complement system [8,9,10] can immediately sense the presence of viral particles (Figure 1a). Complement elements can directly bind to the viral capsid [10], while Toll-like receptors can recognize the pathogen-associated molecular patterns (PAMPs) of adenoviruses [6,11,12]. Within 24 hours after the injection of the Ad vector, the very early toxicity occurs [13] and it is characterized by thrombocytopenia [14] and massive cytokine production [10]. Resident macrophages, such as Kupffer cells (KCs), contribute to the elimination of the injected vector [15,16,17]. In addition to this, the activation of KCs consequent to uptake of Ad vectors results in the production of different chemokines (MIP-2, MPC-1, IP-10 and RANTES) and pro-inflammatory cytokines (IL-1, IL-6 and TNF-α) [15,18]. Cyanosis, dyspnea, lethargy and nausea are, for instance, some of the relevant clinical complications that have been described in non-human primates during the first hours subsequent to the administration of the Ad vector [19].

Activation of the cellular response typically occurs from three to seven days after the injection (Figure 1b). During this phase, the early toxicity is mainly mediated by the activation of cytotoxic T-lymphocytes (CTLs). Viral proteins produced in infected cells are processed and presented as peptides on the major histocompatibility complex (MHC)-I [20]. CTLs recognize these cells and lyse them [21]. In addition, mature CD4^+^ T cells can also contribute to the elimination of adenovirus-transduced hepatocytes [22]. At high viral loads, the liver inflammation can progress to severe hepatic necrosis and systemic inflammatory response syndrome [5].

Late toxicity is the result of mature cellular and humoral immune responses (Figure 1c). Antigen presenting cells (APCs) are activated by uptake of Ad vectors and present Ad-derived peptides on MHC-II to CD4^+^ T lymphocytes, leading to B cell activation. The subsequent production of neutralizing antibodies (NAbs) results in a fast clearance of Ad vector and it leads to the creation of an immunological memory [23]. Weeks later, the host immune system can recognize the transgene expression. Elimination of transduced cells causes the loss of transgene expression [24]. 

## 2. Reducing the Immunogenicity of Ad Vectors

The toxicity caused by Ad vectors is the result of different immunological mechanisms: (i) the inflammation in response to the replication of the Ad vectors and to the production of viral proteins; (ii) the innate immune response which is not related to virus replication and is dose-dependent [13,17,18,19,25]. Different strategies have been developed to reduce the immunogenicity of Ad vectors.

### 2.1. Genetic Modifications

Adenoviruses (Ads) feature two main groups of genes named *early* (*E*) and *late* (*L*) genes. *Early* genes are necessary for virus DNA replication and for controlling the host cell metabolism, while *late* genes encode for all the structural proteins necessary for the capsid [26]. 

First-generation Ad (FG-Ad) vectors feature the deletion of *E1* and/or *E3* genes and, for this reason, they are unable to replicate inside “normal” cells. More specifically, *E1A* and *E1B* gene products are able to block the activity of p53 and p73 proteins [27]; in addition to this, *E1A* is also able to promote cell proliferation by repressing retinoblastoma (Rb) protein activity [28]. The adenovirus *E3* region features nine different genes and is not essential for viral replication [29]. Nevertheless, four of these genes potentially encode for proteins which seem to have immunomodulatory effects [30]. Among these proteins, adenovirus E3-gp19k protein is a transmembrane glycoprotein responsible for retaining MHC-I molecules in the endoplasmic reticulum (ER) [31], thus evading recognition of infected cells by cytotoxic T cells (CTLs) and promoting virus persistence inside the host [32]. However, in spite of these deletions, FG-Ad will still have most parts of the *E* and *L* genes. The “leaky” expression of these genes leads to the production of viral proteins even without an active replication. Infected cells process these proteins and present them as peptides to MHC-I and MHC-II molecules, causing the engagement of cytotoxic T lymphocytes (CTLs) [20,33,34]. 

Second generation Ad (SG-Ad) vectors combine deletions of *E1* and *E3* with deletions in *E2* or *E4* regions [35]. The reduced number of viral genes results in a decreased background production of viral proteins. In addition to this, the higher number of deletions results in an increased space for the insertion of bigger expression cassettes [36].

The most advanced Ad vector is devoid of all viral genes, except for the two inverted terminal repeats (ITRs) and the packaging signal (*psi*). These vectors are also called Helper-dependent Ad (HD-Ad) vectors because their production requires the use of a Helper virus (HV). The HV is an *E1*-deleted serotype 5 Ad (Ad5) that provides in *trans* all the other viral proteins that are necessary for the rescue of the HD-Ad vector. 293Cre4 cells, encoding for the viral gene *E1* and for the recombinase CRE, are used for the amplification of HD-Ad vectors in order to decrease HV contamination. In fact, the recombinase CRE recognises the *loxP* sites that surround the *psi* signal, causing its elimination from the HV’s genome. Lacking the *psi* packaging signal, the HV’s genome cannot be packed in the virions [37,38,39]. The administration of HD-Ad leads to decreased early and chronic toxicities, thus they are less immunogenic than FG-Ad and SG-Ad [5,25,40,41]. The enhanced safety profile of HD-Ad vectors makes them the safest Ad vectors available nowadays for gene therapy. 

Impairing the replication of viral vectors has not solved the problem related to the innate immune response. In fact, the immune system still can sense HD-Ad infection through TLRs, the complement system [6] and resident macrophages, such as Kupffer cells [16]. For this reason, it is necessary to modify the capsid in order to reduce the interactions with the elements of the immune system. Recently, Coughlan and colleagues have studied an Ad5 vector with a mutated fiber knob domain that displays a decreased binding to coagulation factors and complement factors. This vector does not agglutinate human erythrocytes and fails to cause thrombocytopenia after intravenous delivery; furthermore, it also shows a decreased induction of pro-inflammatory cytokines, leading to a low-level toxicity (aspartate aminotransferase/alanine aminotransferase) if compared with normal Ad5 vectors [42]. Seregin and colleagues have engineered an Ad5 vector to display the human complement inhibitor decay-accelerating factor (DAF) on the *C*-terminus of Ad capsid protein IX. It was reported that a lower activation of the complement alternative pathway resulted in a reduced vascular endothelium activation, a lower natural killer (NK) cells induction, a decreased cytokine production and a less pronounced thrombocytopenia [14]. 

### 2.2. Improving the Safety Profile by Chemical and Non-Chemical Modifications

**Figure 2:**
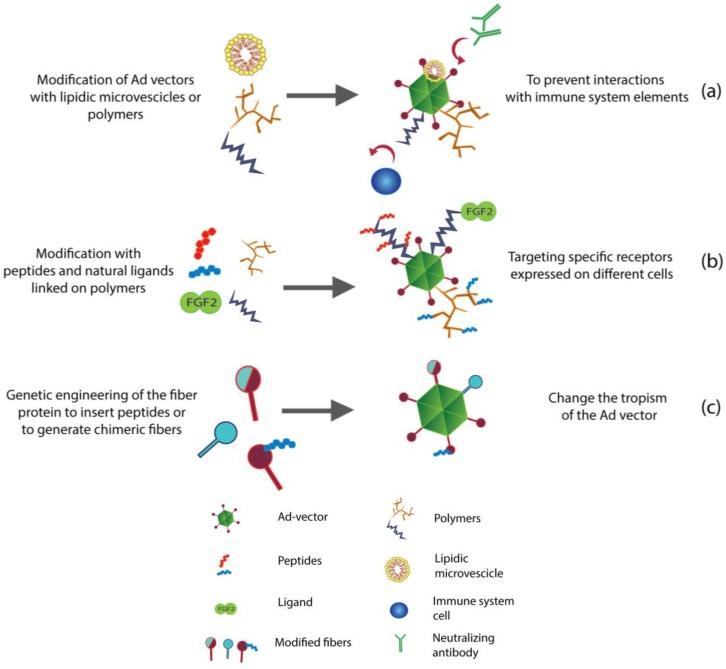
Engineer the surface of Ad vectors. (**a**) Conjugation of polymers and lipidic microvescicles to the surface of Ad vectors results in a reduced immunogenicity and an increased persistence of Ad vectors in the blood stream; (**b**) Moreover, polymers can also be modified with peptides and ligands to target specific receptors, thus allowing efficient re-targeting; (**c**) Fibers can be genetically modified to change the tropism of Ad vectors. Chimeric fibers can be generated using shaft and knob domains from different adenovirus serotypes. In addition, fibers can be engineered to display peptide motifs that target specific receptors or integrins.

As highlighted above in this review, most of the very early toxicity elicited by Ad vectors is caused by the interactions between the capsid of the virus and specific immune sensors called pattern recognition receptors (PRRs). To soften these interactions, different strategies to shield the capsid have been explored (Figure 2a). 

#### 2.2.1. PEGylation as a Tool to Reduce the Immunogenicity

Polyethylene glycol (PEG) is a commonly used polymer that is able to increase the half-life of drugs and to reduce their immunogenicity [43]. PEGylation has been successfully applied to Ad vectors, leading to changes in transduction efficiency [5,44,45] and to a prolonged circulation time of injected vectors [44]. Furthermore, PEGylation of Ad vectors is also able to lower the activation of the immune system [46] as proved by lower levels of pro-inflammatory cytokines (IL-6, IL-12 and TNFα) [5]. Intravenous injection of PEGylated Ad vectors results in a significantly decreased priming of Ad-specific cytotoxic T lymphocytes in comparison with unmodified Ad [47]. Eto and colleagues have reported a reduced production of anti-Adenovirus antibodies (anti-Adv Abs) *in vivo* after the injection of PEGylated Ad [48]. Moreover, PEGylation has proved to be able to protect Ad vectors from existing Nabs as well [5].

#### 2.2.2. New Generation of Biomaterials for Less Immunogenic Ad Vectors

Advances in the biomaterial field have enabled the use of new generations of polymers in order to engineer the surface of Ad vectors. Cationic polymers can be used in combination with PEG. For instance, PEG-poly-l-lysine, a multi-block copolymer based on poly(l-lysine) and PEG, can be used to coat Ad vectors, showing low toxicity *in vitro* and biodegradable properties [49]. Similarly, Zeng and colleagues have shown that coating Ad vectors with a cationic PEG derivative (APC) results in the protection of the vector from NAbs. In fact, the exposure to NAbs does not ablate the transduction activity of ACP-coated Ad vectors *in vitro* [50]. The N[2-hydroxypropyl] methacrylamide (HPMA) copolymer has also been used for coating Ad vectors. The HPMA coating leads to an increased half-life, to a lower NAbs binding and to a decreased interaction with complement receptor 1 (CR1) on human erythrocytes [51]. Therefore, the protection of vectors from NAbs due to a HPMA copolymer coating results in an efficient transduction of these vectors to CAR-positive and CAR-negative cells, even in the presence of NAbs [52]. Arginine-grafted bio-reducible polymers (ABPs) can also be conjugated to the surfaces of Ad vectors in order to decrease the production of pro-inflammatory cytokines. In fact, ABP conjugation to an oncolytic Ad significantly lowers IL-6 production *in vitro* [53] and *in vivo* [54].

Beside polymers, lipids have also been used to improve the safety profile of viral vectors [55]. Cationic liposomes are artificially generated lipid vesicles frequently used for DNA or drug delivery. Ad vectors can be complexed to small unilamellar vesicles (SUV) by a simple mixing reaction (30 min at 37 °C) [56]. In mice injected with Adenovirus/SUV complexes, the levels of anti-adenoviral vector antibodies are 6.5-fold lower than those from mice injected with the naked vector; therefore, these polymer-coated vectors are less immunogenic. Moreover, SUV-conjugated Ad vectors are less susceptible to inactivation by neutralizing antibodies, thus allowing the successful re-administration of the adenoviral vector to mice [56]. 

Lipidic envelopes can also be used to shield Ad vectors in order to lower the immune response. DOTAP:CHOL and DOPE:CHEMS are, respectively, cationic and anionic lipid envelopes able to decrease the production of NAbs after intra-venous injection [57]. 

## 3. Re-Targeting of Ad Vectors

Coxsackievirus and adenovirus receptor (CAR) has been described as the receptor on the cell membrane for the subgroup C and B adenoviruses (Ads) [58,59]. After the initial interaction between CAR and the fiber protein of the capsid, the engagement of αv integrins with the penton base leads to the internalization of Ads [60]. However, Ads can also take advantage of a CAR-independent mechanism that involves different coagulation factors, such as coagulation factor X (FX), coagulation factor IX (FIX) [61] and the complement factor C4BP [62]. The Vitamin K-dependent FX is positively charged, thus it is able to bind the negative hexon protein on the surface of Ads. More specifically, the serine protease domain of the FX interacts with the hypervariable regions (HVRs) of the hexon protein. Having the FX on their surface, Ads can then interact with the heparan sulfate proteoglycans (HSPGs) on the cellular membrane of hepatocytes [63]. The ablation of the formation of functional FX, by warfarin administration prior to Ad vector injection, leads to a decreased infection of the liver [64]. This model has been confirmed in non-human primates [65]. Similarly, FIX and C4BP bind to the fiber of Ads, promoting interactions with HSPGs and low-density lipoprotein receptor-related protein (LRP) [62]. Based on this knowledge, different strategies have been developed to re-target Ad vectors to different tissues and to avoid liver pooling. 

### 3.1. Genetic Engineering to Re-Target Ad Vectors

To de-target Ad vectors efficiently, Mizuguchi and colleagues have generated two differently mutated Ad5s: one unable to bind CAR due to a mutation in the fiber, and the second one unable to bind αv integrins because of a mutation in the penton base. Consistent with the CAR-dependent model, the mutated Ad vectors show reduced transduction *in vitro* when using CAR positive cells. However, *in vivo* experiments show no significant difference in liver transduction between modified and non-modified Ad vectors [66]. These findings have therefore revealed the presence of an alternative mechanism for the liver tropism of Ad vectors and their entry into target cells. As described in the previous section, FX seems to be crucial for the transduction of hepatocytes and for liver accumulation of Ad vectors. Alba and colleagues have generated an Ad vector unable to bind FX due to a mutated hexon protein [67]. This modification significantly decreases the transduction of liver, causing a completely different biodistribution profile of Ad vectors [63]. 

Controlling the replication of viruses/vectors also represents a valid approach in order to control their target. Conditionally replicating Ads (CRAds) are able to replicate only in certain cell types. For instance, oncolytic adenoviruses (OAds) are able to replicate only in tumor cells. Such selectivity could be achieved by impairing the activity of viral proteins that are necessary for taking the control of cellular metabolism. OAds that lack the activity of E1A or E1B proteins are not able to block p53 or Rb inside the infected cell. The inability to block p53 and Rb results in no viral replication. Nevertheless, if the infected cell shows defective p53 or Rb pathways, such as a tumor cell, the viral replication can proceed leading to the lysis of infected cells [68,69,70,71,72]. However, this kind of oncolytic vectors are clearly limited by the pathways that are eventually disrupted inside tumors cells. A more accurate approach is represented by the use of tumor-specific promoters, to be used to drive the replication of the viral genome. Rodriguez and colleagues inserted the prostate-specific antigen (PSA) promoter upstream of the Ad *E1A* gene in order to restrict the viral replication to prostate cancer cells [73]. Similarly, Kim and colleagues used the human telomerase promoter hTERT [74] to regulate the expression of *E1A* viral gene. Targeting the tumor microenvironment is also possible. Several solid tumors develop and hypoxic environment. Therefore, hypoxia-responsive elements (HRE) have been successfully used to allow virus replication in hypoxic cells but not in normoxic cells [75]. 

#### 3.1.1. Use of Chimeric Fibers to Re-Target Ad

Generation of fiber-mutated vectors represent a successful strategy to re-target Ad vectors. Shaft, knob and base domains of fibers from different serotypes can be combined to create chimerc fibers (pseudotyping) (Figure 2c). Zhao and colleagues have constructed a serotype 5 Ad (Ad5) having a fiber composed by the knob and shaft domain of Ad35 and the base domain from Ad5 (Ad5/35). This modification resulted in an increased infectivity of bladder cancer cells in comparison with normal Ad5 *in vitro* [76]. Consistently, Shayakhmetov and colleagues have reported a decrease in the transduction of hepatocytes *in vitro* when using Ad5/35. Nevertheless, efficient liver de-targeting could not be achieved *in vivo* due to the effect of coagulation factors. Only in absence of blood, liver transduction of Ad5/35 was significantly lower than of Ad5 [62]. Granio and colleagues have reported efficient delivery of CFTR gene to cystic fibrosis primary airway epithelia by using Ad5/35 [77]. Similarly, Diaconu and colleagues used an engineered Ad5 featuring the complete fiber of Ad19p. In addition to this, the fiber had been modified in order to have kidney vascular targeting moieties placed into the knob HI loop region. These modifications resulted in an improved tumor-to-liver ratio in comparison with Ad5 [78]. Rodriguez and colleagues focused on improving the intestinal transduction of Ad5 vectors. Ad40 shows intestinal tropism; hence, Ad5 vectors pseudotyped with the short fibers of Ad40 showed an increased infection of colon in comparison with Ad5 vectors [79]. 

As stated above, the binding of Ad vectors to HSPGs is important for liver transduction. Interestingly, different groups have reported that the length and the flexibility of the fiber, as well as the presence of the KKTK motif in the shaft domain, could affect HSPGs binding and, as a result, liver tropism too [80,81,82,83,84,85]. Serotype 3 Ad (Ad3), a species B Ad, features a shorter shaft domain in comparison with Ad5 [86] that lacks the KKTK region of the third repeat [87]. For this reason, Ad3 could be less dependent on HSPGs binding. Moreover, it is reported that Ad3 could use CD46 as a receptor [88], a molecule overexpressed in different tumors [89,90]. Murakami and colleagues have shown that pseudotyping Ad5 with the fiber knob domain of Ad3 (Ad5/3) resulted in an increased transduction of prostate tumor cells *in vitro* [91]. Kim and colleagues have generated a conditionally replicative version of Ad5/3 by deleting 24 bases in the *E1A* gene, thus making the vector able to replicate only inside tumor cells that show a disrupted Rb pathway. Ad5/3-D24 show a remarkable safety profile during pre-clinical studies in hamsters [92] and during a phase I clinical trial in patients with recurrent ovarian cancer [93]. Van de Ver and colleagues have investigated whether Ad5/3 could be an efficient tool for transducing dendritic cells (DCs) *ex vivo*. They have shown that an accurate *ex vivo* transduction of DCs is possible due to the interaction between Ad5/3 and B7 molecules (CD80 and CD86) when expressed on mature myeloid DCs from human skin [94]. Recently, Koski and colleagues have studied a mutated version of Ad5/3 *in vivo*. The fiber of this vector features a KKTK-deleted shaft domain of Ad5 and the knob domain of Ad3. Interestingly, while liver de-targeting had been achieved, transgene expression in tumors was nearly abolished, highlighting that the accumulation of Ad vectors is not a sufficient condition for transgene expression [95]. These results seem to be consistent with another study that has shown how Ad vectors, modified not to interact with HSPGs and CAR, fail to transduce efficiently tumors and other tissues in comparison to wild type Ad vectors [96].

#### 3.1.2. Insertion of Peptides and Other Ligands in the Structure of Ad Vectors

Fibers of Ad vectors can be modified to present small peptides, ligands, or parts of antibodies. These kinds of modifications provide the opportunity to target cells that are expressing or overexpressing specific receptors. Belousova and colleagues have generated an Ad5 vector targeted to human epidermal growth factor receptor 2 (Her2)-positive cells through the incorporation into the fiber of an affibody, which is specific for Her2. This re-targeted vector was able to transduce Her2 positive breast cancer cells, along with consistent and significant decrease in transduction of Her2 negative cells in comparison with normal Ad5 [97]. Targeting Ad to integrins, which are often overexpressed by tumor cells [98], has proven to be a successful method to enhance tumor uptake of vectors. Coughlan and colleagues have generated an Ad5 vector with an αvβ6 integrin targeting motif in the HI loop of knob domain. This Ad vector shows a remarkable increase in CAR-independent transduction and cytotoxicity of αvβ6 positive carcinoma cell lines. *In vivo* experiments have confirmed an improved tumor uptake following systemic delivery, as well as a decreased liver transduction [99]. Ad vectors can also be targeted to integrins by the insertion of an RGD motif in the HI loop of the fiber knob domain. The insertion of the RGD motif in this region results in an increased transduction of human bladder cancer cell lines if compared with normal Ad5 [43]. 

Pesonen and colleagues have tested the oncolytic Ad5-D24-RGD vector, armed with granulocyte-macrophage colony stimulating factor (GM-CSF), achieving promising results in human patients with advanced chemotherapy refractory solid tumors [100]. Rojas and colleagues have shown that the incorporation of the RGD motif in the fiber shaft domain, instead of the HI loop, leads to a decreased liver toxicity and to an improved antitumor activity [101]. Interestingly, Mastui and colleagues have shown that RGD motif can be inserted in both FG and HI loops of the fiber. In fact, a pseudotyped Ad5F35 loaded with RGD motifs in both loops show greater transduction efficacy of CD46-negative cells due to increased integrin targeting [102]. Gamble and colleagues have also used the double RGD-loading approach to enhance successfully the cytotoxic activity of an oncolytic Ad5 [103]. Wu and colleagues reported that the hexon protein could also be modified in its hyper-variable regions (HVRs). They have shown that the insertion of His_6_ epitopes in different HVRs of the hexon does not affect the virion stability and the CAR-mediated Ad5 native infectivity *in vitro* [104]. RGD and other peptide motifs can be inserted as well in the hexon HVRs. Recently, Di and colleagues showed that also the NGR motif (which binds to endothelial CD13) or the HIV-derived Tat PTD peptide can be successfully inserted in the hexon HVR5 region. In particular, the insertion of the Tat PTD in the hexon results in an increased transduction of the CAR-negative cell lines A172 and CHO-K1 in comparison to the unmodified Ad5 [105]. 

### 3.2. Chemical and Non-Chemical Modifications to Re-Target Ad Vectors

The PEGylation of Ad vectors has proven to be a reliable strategy that can reduce the immunogenicity of Ad vectors. In this section, we would like to highlight that coating the Ad vectors with either PEG or the new generation of polymers can also modify their biodistribution, as well as their transduction efficacy. 

#### 3.2.1. Lipid Envelopes for Liver De-Targeting

The construction of lipid envelopes in order to engineer the surfaces of Ad vectors could modify their transduction efficacy. Cationic envelopes represent an interesting example of how important is to preserve the intra-cellular trafficking of Ad vectors. Positive enveloped Ad vectors can bind to the negatively charged cell membrane, but the presence of the envelope disrupts normal endosomal trafficking leading to no transgene expression *in vitro* [56]. Cationic non-pH sensitive envelopes (DOTAP:CHOL) and anionic pH-sensitive envelopes (DOPE:CHEMS) have been successfully used *in vivo* to decrease liver uptake. Interestingly, these two envelopes do not completely ablate the *in vitro* viral uptake in hepatocytes. Differences in electrostatic interactions between enveloped Ad vectors and the negative cell membrane seem to be responsible for the different viral uptake *in vitro* [57]. In addition to this, artificially enveloped Ad vectors have shown an increased ability to penetrate into tumor spheroid cell culture [56].

#### 3.2.2. PEGylation as a Liver De-Targeting Tool

As reported by different research groups, PEGylation of Ad vectors seems to reduce the transduction of cells *in vitro* [106,107,108]. Interestingly, *in vivo* experiments have shown that the transduction of liver and the biodistribution of Ad vectors are affected by the size of PEG molecules used. In fact, no significant changes have been observed in the biodistribution profile of Ad vectors PEGylated with a low molecular weight PEG [107] while Ad vectors PEGylated with the high molecular weight PEG have shown reduced liver transduction [106]. 

The surface of Ad vectors can drastically change depending on the size of PEG particles, leading to the alteration of the transduction efficiency of cells *in vitro* and *in vivo*. The anatomical, histological and physiological species differences should be taken into account when using PEGylated vectors: for instance, PEGylation does not compromise hepatic gene transfer in rodents [5,107,109] while it is able to lower the transduction efficiency of Ad vectors in non-human primates [45]. Different factors could contribute to explain these findings: (i) non-human primates feature a smaller size and a lower density fenestrate endothelium in the liver in comparison to rodents and this could affect the ability of coated-Ad vectors to reach the hepatic tissue [45]; (ii) coating Ad-vectors reduces the interactions with the coagulations factors [108,110] which proved to be important for the transduction of hepatocytes; (iii) expression of CAR is lower in the liver of non-human primates with respect to their rodent counterparts [111].

These interesting results can also be explained by the non-specific nature of the PEGylation process. PEG molecules can cover areas of the capsid that are necessary for cell entry (e.g., fibers). Matsui and colleagues developed a more accurate PEGylation process by using the FX as an adapter in order to control the coating. They have shown that the hexon-specific PEGylation did not substantially disturb the interaction between the fiber protein and the CAR receptor. Interestingly, they have also reported that the hexon-specific PEGylated Ad vectors feature an increased transduction efficacy of CAR-negative cells in comparison to unmodified Ad vectors [112]. Similarly, Suzuki-Kouyama has inserted a biotin-binding peptide (BAP) in the HVR5 of the hexon, and PEG was then specifically conjugated to the HVR5 via avidin-biotin interaction [113]. 

#### 3.2.3. Liver De-Targeting by New Generation of Polymers

Green and colleagues have reported that coating vectors with HPMA polymer result in a decreased liver transduction in comparison with un-coated Ad vectors. The HPMA-coated Ad vectors also feature prolonged half-life and bioavailability [114]. Wang and colleagues have proposed chitosan as an alternative material to coat Ad vectors, showing that it can enhance the transduction of corneal epithelial cells *in vitro* [115]. 

Successful liver de-targeting of Ad vectors has been achieved by using cationic polyamidoamine (PAMAM) cascade dendrimers. *In vivo* imaging tests have confirmed the successful liver de-targeting of the Ad vectors coated with PAMAM dendrimers [116]. This new class of polymers feature a regular dendritic branching and radial symmetry and they have already been studied as a new delivery system for gene therapy [117]. Ad vectors coated with PAMAM dendrimers are protected from NAbs and can be re-targeted to other tissues or tumors by adding targeting ligand on the polymer [118]. 

#### 3.2.4. Re-Targeting with Microbeads or Magnetic Particles (Mags)

Mags and microbeads are attractive tools for vector re-targeting. Pandori and colleagues have described how Ad vectors can be conjugated to silica microbeads in order to enhance transduction of different target cells *in vitro.* Different mechanisms could explain the enhanced transduction efficiency: (i) the accumulation of Ad particles on the beads could increase the number of viral particles on/near the cell membrane, favouring the interactions with viral receptors; (ii) the presence of such structures (Ad/microbeads) could promote also a receptor-independent uptake [119]. Similarly, Mags can be complexed to Ad vectors for *in vitro* or *ex vivo* cell engineering. During the infection, an external magnetic field is used in order to accumulate the Ad-Mag complexes on the target cells. Sapet and colleagues have shown that this approach improves the transduction efficacy in different cell lines (C6, COS-7, NIH-3T3 and HEK-293T cell lines) including neurons (DIV 7 cell line); hence, lower doses of vectors are required for gene transfer. Moreover, Ad-Mag complexes have been able to increase the transduction efficacy of non-permissive or low CAR-expressing cells lines. These findings suggest that Ad-Mag complexes could use a CAR-independent entry mechanism or benefit from the concentration of viral particles onto target cells due to the external magnetic field [120]. 

#### 3.2.5. Accurate Re-Targeting of Ad Vectors with Peptide Motifs and Ligands

The availability of high-throughput screening methods has enabled the identification of peptides that are able to target tumor cells or tumor neo-vasculature. For this reason, these peptides, called tumor-homing peptides, may be used to drive viral vectors towards the tumor site. 

For example, the CGKRK peptide is able to accumulate on the surface of tumor vessels, showing an association with the tumor neo-vasculature [121]. Hence, the CGKRK peptide has been tested for its ability to re-target PEGylated adenoviral vectors: PEG molecules are conjugated to the surface of adenoviral vectors; then, by a chemical reaction, the CGKRK peptide is conjugated to the functional group of PEG [122] (Figure 2b). Yao and colleagues have tested the transduction efficacy of the Ad-PEG-CGKRK vector into B16BL6-bearing mice. They found an increased amount of Ad-PEG-CGKRK in tumor tissue if compared with Ad and Ad-PEG. In addition to this, they have reported a lower amount of Ad-PEG-CGKRK vector in liver tissue in comparison with normal Ad vector, proving that the liver de-targeting ability of PEG had been preserved even after the conjugation with the targeting peptide [123]. Tumor-homing peptides can also be used to create viral vector-based imaging systems. We have already described the tumor-homing properties of RGD peptides, which are based on the recognition of integrins that are often overexpressed on many different tumors. Xiong and colleagues have used a luciferase-expressing Ad vector coated with RGD-PEG molecules and, as a result, the radiolabelling with positron emitter radioisotopes had enabled them to visualize *in vivo* the accumulation of the vector in tumors [124]. Cyclic-RGD peptides can be used to improve the transduction efficacy of Ad vectors *in vitro*. Human mesenchymal stem cells can be efficiently transduced by using a GFP-expressing Ad vector in presence of cyclic-RGD peptides [125].

To achieve accurate targeting of tumors, Ad vectors need to recognize specific molecules on the membrane of tumor cells. Several receptors, such as EGFR or FGFR, are commonly overexpressed in tumors. For this reason, real ligands, such as epidermal growth factor (EGF) and fibroblastic growth factor (FGF), can be studied for tumor targeting [126]. Berg and colleagues have used the poly(2-(dimethylamino)ethyl methacrylate) (pDMAEMA) polymer as a linker among EGF, the Ad vector reporting successful transduction of EGFR positive cells, and CAR-deficient cells. Interestingly, the light treatment of infected-target cells could enhance transduction efficiency of EGFR-targeted viral complexes. In fact, a physical technology, termed photochemical internalization (PCI) can enhance transgene expression. Although, it is unclear how PCI can exactly enhance the transduction efficacy of Ad vectors, several hypotheses have been proposed: (i) light activation of amphiphilic photosensitizers that localize in endocytic vesicles can enhance endosomal escape of internalized Ad vectors [127]; (ii) Ad particles released by PCI might be less prone to degradation of the DNA during translocation to the nucleus [128]; (iii) irradiation of target cells could result in an increased expression of adenovirus encoded-transgenes [129]; (iv) CAR and integrins could also be involved in PCI-mediated enhanced transduction [130]. Similarly, Seymour and colleagues have conjugated murine EGF to reactive HPMA copolymer and the mEGF-HPMA has been used to coat the Ad; this polymer-coated virus has shown selective and enhanced transduction into EGFR positive cells *in vitro*. Consistently, an oncolytic Ad vector coated with mEGF-HPMA had significantly longer median survival if compared with mice that received untargeted HPMA-Ad [131]. 

An EGF mimetic peptide (GE11) has been used to re-target dendrimer-coated Ad vectors. This peptide has been linked to the PAMAM dendrimer polymer through a PEG linker and then the adenoviral vector has been coated with the PAMAM-PEG-GE11 complex. *In vitro* transduction assays had shown that the re-targeted vector was able to increase the transgene expression in target cells if compared with the untargeted vector [118]. 

Folate receptor, like EGFR, is expressed by different types of carcinomas, such as ovarian cancer [132], nasopharyngeal and laryngeal carcinoma [133]. A recent study has described the *in vivo* performance of a chitosan-PEG-folate coated oncolytic Ad. As expected, the presence of PEG increased the blood circulation time of oncolytic Ad-nanocomplexes, with a remarkable 378-fold decrease in liver uptake if compared with naked Ad. In addition to this, an outstanding tumor-to-liver ratio was observed, resulting in an improved anti-tumor response [134]. 

Wang and colleagues have developed a ligand-based re-targeting approach by using the fibroblastic growth factor 2 (FGF2) molecules to target FGFR positive malignant cells. The Ad vector has been directly conjugated to FGF2 and tested *in vivo* using either CAR positive or CAR negative cell lines in murine xenograft tumor models. They have shown that adenoviral transduction in different CAR-deficient glioma cells were increased when Ad vectors were retargeted to FGFR1. A following *in vivo* experiment confirmed an increased transduction of tumors as proved by GFP expression in the tumor [135]. FGF2 can also be conjugated to reactive polymers (Figure 2b), such as PEG [136] or pHPMA [137], resulting in an increased half-life in blood. Nevertheless, FGF2 coated vector has shown an unexpected binding of erythrocytes [137]. This finding suggests that non-specific interactions may occur *in vivo*, leading not only to a reduced availability of injected vectors, but also to potential health risks and undesirable side-effects.

## 4. Conclusions

The translation of gene therapy research into clinic has proved to be a challenging task. However, the history of the evolution of Ad vectors is a clear example of how limitations can be overcome by the synergistic collaboration of different fields of research. Genetic engineering, immunology, virology and biomaterial sciences have been able to significantly improve the efficacy of Ad vectors over time. 

Avoiding the activation of the immune system is an important requirement for the treatment of genetic disorders in order to prevent acute immune reactions and the loss of transgene expression. The deep understanding of the replication mechanisms led to the generation of replication-defective Ad (RDAds) vectors that show reduced chronic and early toxicities. Nevertheless, these vectors are not able to avoid the activation of the innate immune response. For this reason, a growing number of studies are focusing on the conjugation of Ad vectors with a wide variety of polymers and lipid envelopes. 

Coated-Ad vectors generally feature increased half-life, low immunogenicity and less pronounced liver-tropism. The conjugation of Ad vectors to biomaterials increases the size of vectors and largely modifies the surface of the capsid. Therefore, the consequences might be different from species to species. In spite of these limitations, hiding the Ad vectors from the immune system is still important for the clinical translation of Ad-vector based therapies for genetic diseases, in order to avoid excessive activation of the immune system and to protect the vectors from the pre-existing immunity. For this reason, multispecies studies and further progress in the knowledge of conjugation of biomaterials to Ad vectors can considerably improve the applicability to clinical research of such technology. 

It is worth mentioning that the complete loss of the immunogenicity of Ad vectors does not represent an absolute requirement for all the Ad vector-based therapies. For instance, while an immunogenic Ad vector might not be suitable for gene therapy of genetic disorders, it could represent a valid tool to stimulate the immune system in cancer immunotherapy or Ad-based vaccines. 

In this review, we also reported how Ad vectors can be efficiently re-targeted to different tissues, including tumors, when displaying ligands or peptide motifs on their surface. Nevertheless, the ligand-based re-targeting of Ad vectors might lead to unexpected side effects if the target receptor is present in tissues that are not intended to be targeted. Being limited by information about specific receptors on target cells, this approach will considerably benefit from the increasing knowledge of membrane proteins on different types of cells.

As we reported, the selectivity and the safety profile of OAds rely on the differences between tumor and normal cells, hence on the ability to identify molecular pathways that are specific for tumor cells. For this reason, deepening the knowledge of tumors and their molecular abnormalities would definitely improve the efficacy of OAds and reduce the toxicity due to unwanted viral replication in normal cells. 

The approval of Ad-based drugs for commercial use represents a milestone that generates fresh enthusiasm and lays the foundation for larger investments in the field. Gendicine was the first gene therapy product to obtain the licence for clinical use in humans. It is a p53-expressing Ad vector that was approved by the Chinese State Food and Drug Administration in 2003 for the treatment of head and neck squamous cell carcinoma [138]. In 2006, another Ad-based drug entered the Chinese market: Oncorine. This adenovirus features a deletion in the *E1B 55K* region and it was approved for the treatment of head and neck cancer [139]. More products are expected to enter the market in the near feature, thanks also to the considerable amount of data that have been generated by pre-clinical and clinical studies with adenoviruses during these decades.

The new knowledge acquired through the study of tumors, other tissues and biomaterials flows into gene therapy, leading to the production of new strategies and tools for future challenges. 
